# Characterisation of the Expression of Neurotensin and Its Receptors in Human Colorectal Cancer and Its Clinical Implications

**DOI:** 10.3390/biom10081145

**Published:** 2020-08-05

**Authors:** Shengyang Qiu, Stella Nikolaou, Jie Zhu, Peter Jeffery, Robert Goldin, James Kinross, James L. Alexander, Shahnawaz Rasheed, Paris Tekkis, Christos Kontovounisios

**Affiliations:** 1 Department of Surgery and Cancer, Imperial College, London W2 1NY, UK; s.qiu@imperial.ac.uk (S.Q.); stella.nicolaou@gmail.com (S.N.); j.kinross@imperial.ac.uk (J.K.); drsrasheed@gmail.com (S.R.); c.kontovounisios@imperial.ac.uk (C.K.); 2 Department of Colorectal Surgery, Royal Marsden Hospital, London SW3 6JJ, UK; 3 Department of Colorectal Surgery, Chelsea and Westminster Hospital, London SW10 9NH, UK; 4 Lung Pathology Unit, Airway Disease Infection, National Heart and Lung Institute, Imperial College, London SW3 6LY, UK; j.zhu@imperial.ac.uk (J.Z.); p.jeffery@imperial.ac.uk (P.J.); 5 Department of Cellular Pathology, Imperial College, London W2 1NY, UK; r.goldin@imperial.ac.uk; 6 Department of Metabolism, Digestion, and Reproduction, Imperial College, London W2 1NY, UK; j.alexander@imperial.ac.uk

**Keywords:** colorectal cancer, biomarker, neurotensin

## Abstract

*Introduction*: Colorectal Cancer (CRC) accounts for 9% of cancer deaths globally. Hormonal pathways play important roles in some cancers. This study investigated the association of CRC expression of neurotensin (NTS), NTS receptors 1 and 3 (NTSR1 and NTSR3) and clinical outcomes. *Methods*: A prospective cohort study which quantifies the protein expression of NTS, NTSR1 and NTSR3 in human CRCs using immunohistochemistry. Expression levels were then compared with clinico-pathological outcome including histological grade, overall survival (OS) and disease-free survival (DFS). *Results*: Sixty-four patients were enrolled with median follow-up of 44.0 months. There was significantly higher expression of NTS in cancer tissue in CRC with higher T stages (*p* < 0.01), N stages (*p* = 0.03), and AJCC clinical stages (*p* = 0.04). There was significantly higher expression of NTS, NTSR1 and NTSR3 in cancer tissue compared to surrounding normal epithelium (median H-score 163.5 vs 97.3, *p* < 0.01). There was significantly shorter DFS in individuals with CRC with high levels of NTS compared to lower levels of NTS (35.8 months 95% CI 28.7–42.8 months vs 46.4 months 95% CI 42.2–50.5 months, respectively, *p* = 0.02). Above median NTS expression in cancer tissue was a significant risk factor for disease recurrence (HR 4.10, 95% CI 1.14–14.7, *p* = 0.03). *Discussion*: The expression of NTS and its receptors has the potential to be utilised as a predictive and prognostic marker in colorectal cancer for postoperative selection for adjuvant therapy and identify individuals for novel therapies targeting the neurotensinergic pathways. *Conclusions*: High NTS expression appears to be associated with more advanced CRC and worse DFS.

## 1. Introduction

Neurotensin (NTS) is a 13 amino acid peptide which was first isolated in 1973 from bovine hypothalamus and digestive tract [1]. In humans, it is a neurotransmitter in the central nervous system and a hormone in the periphery. It modulates gut function, vascular smooth muscle activity, gastrointestinal motility, and pancreaticobiliary secretions [2,3]. Its physiological actions are mediated through a G-protein coupled receptor, NTS receptor 1 (NTSR1) whilst a second subtype, NTS receptor 2 (NTSR2) has mainly been identified in the central nervous system [4]. The third NTS receptor, NTSR3 is identical to sortilin, a 100 kDa protein with a single transmembrane domain [5]. Recently, NTS and its receptors have been implicated in the carcinogenesis of a broad range of human cancers including colorectal cancers (CRC) [6,7,8,9,10].

Colorectal cancer (CRC) is one of the most common cancers globally, accounting for 9.2% of all cancer deaths [11,12]. CRC survival outcome is strongly influenced by the histological characteristics of the cancer. Traditional prognostic markers include the American Joint Committee on Cancer (AJCC) tissue (T) stage, node (N) stage, grade of differentiation, presence of lymphovascular invasion (LVI), extramural vascular invasion (EMVI), perineural involvement (PNI), inflammatory response, and clear resection margins. Molecular genetic markers in regular clinically use include mutations in KRAS, NRAS, PI3K, PTEN, BRAF and CDX2. The presence of microsatellite instability (MSI) and loss of MLH1, MLH2, MLH6, and PSM2 are also assessed. Presence and absence of these markers are both predictive and prognostic and are important in the clinical management of CRC [13,14,15,16].

Previous studies have demonstrated higher levels of NTS and NTSR1 in CRC tissue compared to normal colonic epithelium. Additionally, there is incremental levels of NTSR1 mRNA expression in normal colonic epithelium, adenomas, and adenocarcinoma. NTS receptors were identified in 40% of in vivo human colon cancer cell lines [17]. The proportion of NTSR1-positive epithelial cells progressively increased in biopsies taken from colonic epithelium with inflammation, dysplasia, and adenocarcinoma in the context of inflammatory bowel disease [18]. Furthermore, NTSR1 mRNA expression increased as colonic epithelial cells progressed along the adenoma to adenocarcinoma pathway; infiltrating margins and tissue from LVI showed highest intensity of NTSR1 expression [19,20].

The key objectives of this study were to characterise the protein expression of NTS, NTSR1, and NTSR3 in human CRC tissue. This study aimed to ascertain if there are differences in the protein expression of NTS and its receptors in different histological grades of CRC. Lastly, the effect of NTS and NTSR expression and CRC survival outcomes are investigated.

## 2. Materials and Methods

### 2.1. Subjects

The participants were prospectively enrolled between 9 July 2014 and 3 November 2016 from three hospitals in London (United Kingdom). Patients aged 18 and over diagnosed with colonic or rectal adenocarcinoma undergoing curative resection were included. Exclusion criteria included patients with known inflammatory bowel disease or those who were unable to give consent.

The participants were randomly selected and were anonymised. Demographic data, histopathologic diagnosis, cancer stage and clinic data were collected by review of patient electronic records (Cerner Corporation, North Kansas City, MO, USA). The histological characteristics of CRC, overall survival (OS) and disease-free survival (DFS) were collected. Extraction of clinical data took place after immunohistochemical (IHC) quantification. All research team members were blinded to all clinicopathological data.

### 2.2. Immunohistochemical Staining

Paraffin-embedded tissue blocks from each participant were cut into 5 µm-thick sections. One section from each subject was stained with haematoxylin and eosin to assess morphological structures.

Primary antibodies used to target NTS (rabbit anti human NTS polyclonal antibody, epitope: aa24-170, LS-C400910, LifeSpan BioSciences, Seattle, WA, USA), NTSR1 (rabbit anti human NTSR1 cytoplasmic domain polyclonal antibody, epitope: 17 amino acid peptide from 3rd cytoplasmic domain of human NTSR1 between aa260–303, LS-A938, LifeSpan BioSciences, Seattle, WA, USA), and NTSR3 (rabbit anti human NTSR3 polyclonal antibody, epitope: aa607-735, NBP1-89745, Novus Biologicals Europe, Abingdon, UK) were sourced from commercial manufacturers. The primary antibodies were validated with IHC staining of human gastric, colon, lung, bladder or ovarian cancers to guarantee specificity to the intended targets. Western blots were also performed by the manufacturers to ensure the antibodies’ specificity for the epitopes. We performed further internal validation of primary antibodies and their compatibility with IHC protocols using positive control tissues (lung adenocarcinoma and A549 cells which were processed into paraffin sections).

NTS, NTSR1 and NTSR3 positivities in the tissue sections were detected using the Dako EnVision Peroxidase staining method. Sections were deparaffinised in xylene baths then rehydrated in graded alcohols to water. Antigen retrieval for NTS, NTSR1 and NTSR3 was performed by boiling the citrate buffer PH 6.0 in a microwave for 7 minutes. Endogenous peroxidase activity was quenched by incubating the sections with Dako REAL Peroxidase-Blocking Solution at room temperature (S2023, Agilent Technologies, Santa Clara, CA, USA). After wash with distilled water and 0.01M phosphate buffered saline (PBS) pH 7.4, 10% normal goat serum blocking solution was used for 20 minutes at room temperature. Then the sections were incubated with primary rabbit anti NTS polyclonal antibody diluted 1:100 at a concentration of 5 µg/mL, or rabbit anti-NTSR1 polyclonal antibody diluted 1:50 at 20 µg/mL, or rabbit anti-NTSR3 polyclonal antibody diluted 1:50 at 4 µg/mL overnight at 4 °C. After wash with PBS, the sections were incubated with Dako EnVision goat anti-rabbit secondary antibody solution at room temperature (K4003, Agilent Technologies, Santa Clara, CA, USA). After a further wash, the sections were incubated with 3.3′-diaminobenzidine tetrahydrochloride (DAB) chromogen (D5637, Sigma-Aldrich, St. Louis, MO, USA) in peroxide buffer for 10 minutes. Stained antigen sites were detected as a yellow-brown product. Finally, the sections were counterstained with haematoxylin to provide nuclear and morphological details and mounted in DPX mounting medium.

Furthermore, non-specific rabbit IgG polyclonal-isotype control (Ab171870, Abcam, Cambridge, UK) and normal rabbit serum (Dako X0902, Agilent Technologies, Santa Clara, CA, USA) were used in place of the primary antibodies as negative control to ensure there was no non-specific staining of tissue by the EnVision secondary antibodies.

### 2.3. Quantification

**Figure 1:**
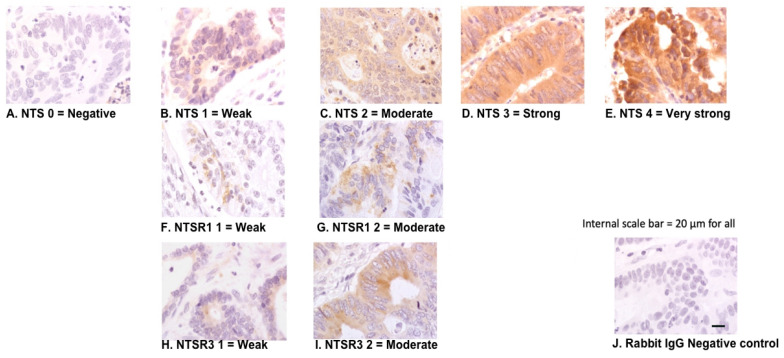
Peroxidase immunohistochemical staining for NTS: (**A**) negative, (**B**) weak signal, (**C**) moderate signal, (**D**) strong signal, (**E**) very strong signal. NTSR1: (**F**) weak signal, (**G**) moderate signal. NTSR3: (**H**) weak signal, (**I**) moderate signal. Rabbit IgG negative control (**J**). Internal scale bar = 20 µm for all.

All quantification was performed on coded slides with the analyst blinded to subject status. Immunostaining intensities and proportions (i.e., protein expression levels) of NTS, NTSR1, and NTSR3 on cancer tissue were quantified using the hybrid-score (H-score) system [21,22]. The protein expression of all 3 markers in the normal surrounding epithelium from each tissue sample were quantified separately. The H-score is based on both the intensity of staining and the proportion of positive cells. The intensity score ranged from 0 to 4 defined as: 0 = negative compared with the background or no specific staining (Figure 1A); 1 = weak detectable staining: light yellow brown in the cytoplasm compared with the stromal background non-specific staining (Figure 1B,F,H); 2 = moderate, readily appreciable staining: yellow brown distinctly marking the epithelial cytoplasm (Figure 1C,G,I); 3 = strong staining: orange brown in the cytoplasm (Figure 1D); or 4 = very strong staining: orange dark brown in cytoplasm (Figure 1E). The areas with staining intensity were “point counted” using an Axioskop 40 light microscope (ZEISS, Oberkochen, Germany) at 200 times magnification and an eye-piece graticule containing 100 crosses [23]. Points overlying tumour associated stroma were ignored. Point counting gave the percentage of positively stained cells. To take into account within tissue variability, a total of 30 evenly distributed microscopic fields which covered the entire cancer tissue or normal epithelium were counted in each specimen. The final H-score (0–400) for a marker on the specimen was calculated using the following formula: total of the intensity scores (0–4) for all viable grid points in 30 fields ÷ total number of viable points in 30 fields × 100.

The within observer variability, expressed as coefficient of variation for three repeat counts of adenocarcinoma epithelial NTS, NTSR1 and NTSR1 positivities in one specimen at different times, ranged between 5% and 6%.

### 2.4. Statistical Analysis

Difference in H-scores of NTS, NTSR1, and NTSR3 between different groups were assessed using the Mann Whitney U test, Kruskal-Wallis test and Wilcoxon matched-pairs test. Chi squared test and Fisher’s exact test were used to compare proportions within categorical data. Spearman’s rank test was used to assess correlation between NTS and NTSR expression, colorectal cancer staging, as well as survival outcomes. Kaplan Meier curves were constructed to compare OS and DFS in participants with below and above median levels of NTS, NTSR1, and NTSR3 expression in cancer tissue and normal epithelium. Log-rank tests was performed to determine survival differences between the groups. Cox-regression was used to identify predictors of disease recurrence. *p* values of less than 0.05 were considered statistically significant.

## 3. Results

Sixty-four patients were enrolled between 9th July 2014 and 3rd November 2016. All participants underwent colorectal surgery, at which time the cancer tissue was resected. Of these, 58 participants’ specimen paraffin blocks were available for immunostaining. All missing data were accounted for: it was not possible to quantify NTS expression in one patient (participant 53) as no tissue section was available for immunostaining. Normal epithelium was not present on the sections for two patients (participant 6 and 30). For one participant, although both cancer tissue and normal epithelium were present on the sections used to stain for NTSR1 and NTSR3, only cancer tissue was present on the section used to stain for NTS.

The median age of participants was 72.2 years (IQR 63.9–82.4 years). There were 30 male patients and 28 female patients. The demographics, operative and postoperative details for the participants are shown in Table 1. The clinical pathological characteristics of the cancers are detailed in Table 2.

### 3.1. Morphological Patterns of NTS, NTSR1, and NTSR3 Protein Expression

**Figure 2:**
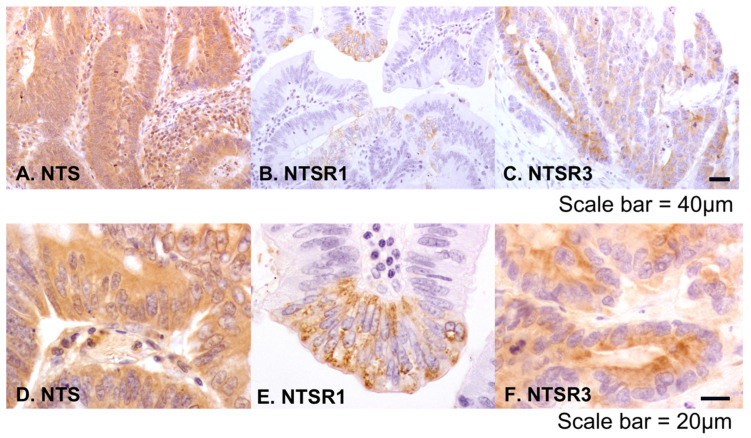
Low magnification peroxidase immunohistochemical staining of colorectal cancer tissue for NTS (**A**), NTSR1 (**B**), and NTSR3 (**C**) at 200 times magnification, internal scale bar = 40 μm. High magnification peroxidase immunohistochemical staining of colorectal cancer tissue of NTS (**D**), NTSR1 (**E**), and NTSR3 (**F**) at 630 times magnification, internal scale bar = 20 μm.

NTS and NTSR3 protein expression was detected in both cancer and surrounding normal colonic epithelium. NTSR1 positivity was observed in cancer epithelium but was negative or very weakly positive in normal epithelium. Within the whole cancer tissue specimens, the distributions of NTS and NTSR3 were localised or diffuse, whereas NTSR1 was focal or patchy. The staining intensities of NTS were from weak to strong (Figure 1B–E) and of NTSR1 and NTSR3 were weak to moderate (Figure 1F–I). An irrelevant primary antibody, rabbit IgG, did not stain the sections (Figure 1J). At the subcellular level, NTS showed cytoplasmic distribution (Figure 2A,D). NTSR1 showed cytoplasmic and granular staining pattern (Figure 2B,E). NTSR3 showed cytoplasmic and membranous patterns of staining (Figure 2C,F).

All CRC tissue and normal epithelium positively stained for NTS. The proportion of cancer tissue which stained positively for NTSR1 (H-score > 0) was significantly higher than the proportion of normal epithelium which expressed NTSR1 (70.7% vs 10.7% respectively, *p* < 0.01, Fisher’s exact test). Similarly, the proportion of CRC tissue which stained positively for NTSR3 was significantly higher than normal epithelium (96.5% vs 80.4% respectively, *p* < 0.01, Fisher’s exact test).

### 3.2. Expression of NTS in Cancer Tissue Was Different between Different Histological Grades

**Figure 3:**
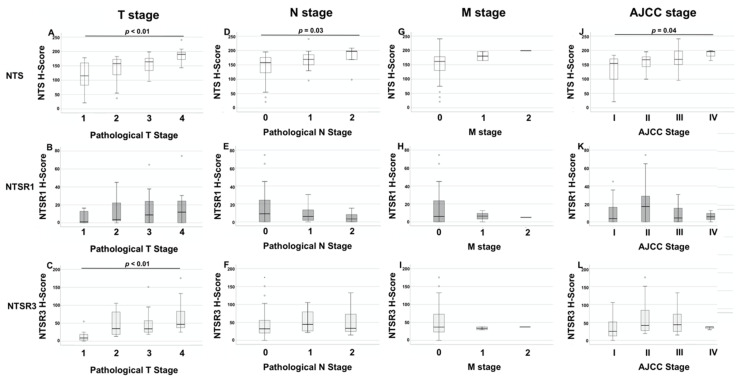
**Column 1:** H-scores of staining of NTS (**A**), NTSR1 (**B**), and NTSR3 (**C**) across American Joint Committee on Cancer (AJCC) pathological T grades. **Column 2:** H-scores of staining of NTS (**D**), NTSR1 (**E**), and NTSR3 (**F**) across AJCC pathological N grades. **Column 3:** H-scores of staining of NTS (**G**), NTSR1 (**H**), and NTSR3 (**I**) across AJCC M grades. **Column 4:** H-scores of staining of NTS (**J**), NTSR1 (**K**), and NTSR3 (**L**) across AJCC clinical stages for colorectal cancer. Solid line: median, ends of boxes: inter-quartile range, error bars: range, circles and asterisks: outliers. Significant *p* values shown for Independent samples Kruskal-Wallis test. Circle: Outliers (values between than 1.5–3 times inter-quartile range). Asterix: Extreme values (values more than 3 times the inter-quartile range).

The H-scores for the expression of NTS, NTSR1, and NTSR3 for each pathological T stages are shown in Table 3. There were significant differences between the intensity of NTS staining in cancer tissue of different T stages, i.e., cancers with different levels of invasion (*p* < 0.01, Independent sample Kruskal-Wallis Test). Higher levels of NTS were seen in cancers with higher T stages. There were also significant differences in the expression of NTS between individual T stages: T1 vs T3 (*p* = 0.03, Mann Whitney U test), T1 vs T4 (*p* < 0.01), T2 vs T4 (*p* < 0.01) and T3 vs T4 (*p* < 0.01) (Figure 3A). There was a significant positive correlation between the cancer T stage and expression of NTS in cancer tissue (R = 0.50, *p* < 0.01, Spearman’s rank test), as well as the expression of NTS in normal surrounding epithelium (R = 0.40. *p* < 0.01).

The H-score of NTS staining in cancer tissue was also significantly different between different N stages (*p* = 0.03, Independent samples Kruskal-Wallis test) and AJCC clinical stages (*p* = 0.04) (Figure 3D,J).

There was a significant difference in the expression of NTSR3 between the different T stages (*p* < 0.01, Independent samples Kruskal-Wallis test) (Figure 3C). There was positive correlation between expression of NTSR3 in cancer tissue and the T stage (R = 0.44, *p* < 0.01, Spearman’s rank test), as well as the expression of NTSR3 in normal epithelium (R = 0.33, *p* = 0.01). There were no significant differences between the H-scores of NTSR1 and NTSR3 across N, M, and AJCC stages.

There were no significant differences in the level of NTS, NTSR1, or NTSR3 expressed in CRC tissue between cancers from different sites of the colon or rectum.

In terms of other histological characteristics, high levels of MSI were present in six of 26 samples sent for genetic analysis. Significantly lower levels of NTS was found in cancer tissue in the MSI-high (MSI-H) group compared the MSI-low (MSI-L) group (median H-score 143.0 vs 170.3, *p* = 0.02, Mann-Whitney U test).

There was significantly higher level of NTS staining in cancer tissue from CRC which were EMVI positive compared to those which were not (Median H-score 177.2 vs 157.6, *p* = 0.04, Mann-Whitney U test).

There was no association between the staining of NTS, NTSR1, or NTSR3 with the size nor the number of cancerous lesions. Furthermore, there were no differences in H-scores of NTS, NTSR1, or NTSR3 between the degree of the differentiation of the cancer, presence of mucinous cancer, background of serrated adenoma, presence of LVI, and presence of PNI.

### 3.3. Expressions of NTS, NTSR1, and NTSR3 in Cancer and Normal Surrounding Epithelium

There was a significant difference between the median H-score for NTS staining in cancer tissue compared to surrounding normal epithelium (163.5, IQR 133.5–178.3 vs 97.3, IQR 48.1–146.8 respectively, *p* < 0.01, Related-Samples Wilcoxon signed rank test). Similarly, there was significantly higher expression of NTSR1 and NTSR3 in cancer tissue compared to corresponding normal epithelium (*p* < 0.01, Related-Samples Wilcoxon signed rank test).

Significant positive correlation between expression of NTS in cancer and normal surrounding epithelium was observed (R = 0.60, *p* < 0.01, Spearman’s rank test). The expressions of NTSR1 (R = 0.41, *p* < 0.01) in cancer and normal epithelium were significantly correlated. Similarly, and NTSR3 (R = 0.52, *p* < 0.01) in cancer and normal epithelium were significantly correlated.

The expression of NTS and NTSR3 in cancer tissue had a positive correlation which was statistically significant (R = 0.46, *p* < 0.01). Finally, the expression of NTSR1 and NTSR3 also had a significantly positive correlation (R = 0.32, *p* = 0.01).

### 3.4. NTS Expression in Cancer Tissue and Survival Outcomes

The median follow-up was 44.0 months (IQR 40–47 months). At the end of the study, 52 participants were alive and 12 (18.6%) died. Follow-up data was not available for two patients. Forty-five (70.3%) patients were disease free. The average OS was 53.9 months (95% CI 49.5–58.2 months). Average DFS was 40.0 months (95% CI 35.6–44.2 months).

There was significantly lower NTS staining in cancer tissue from individuals who were disease-free compared to those who were not (median H-score 157.8 vs 180.1 respectively, *p* = 0.02, Mann-Whitney U test).

**Figure 4:**
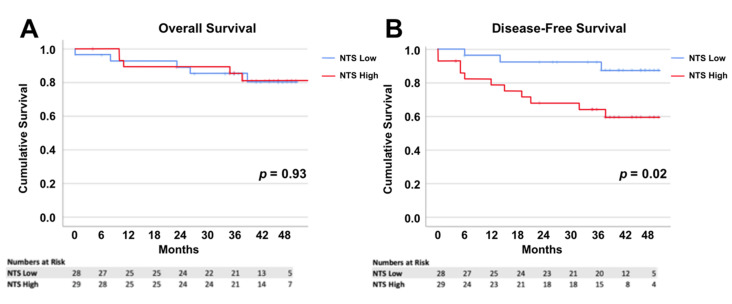
Kaplan Meiers curves of overall survival (OS) of subgroups with high and low expression of Neurotensin (NTS) in cancer (**A**), and disease-free survival (DFS) for subgroups with high and low expression of NTS in cancer (**B**).

There was significantly shorter DFS in individuals with CRC expressing high levels of NTS versus those with cancers with low expression of NTS (35.8 months, 95% CI 28.7–42.8 months vs 46.4 months, 95% CI 42.2–50.5 months respectively, *p* = 0.02, Log rank test) (Figure 4B). There was no difference in OS between these two groups (54.4 months, 95% CI 48.1–60.7 months vs 44.2 months, 95% CI 39.2–49.2 months, *p* = 0.93, Log rank test) (Figure 4A). Above-average NTS expression in cancer tissue was a significant risk factor for disease-recurrence (Hazard Ratio 4.10, 95% CI 1.14–14.7, *p* = 0.03, Cox proportional regression).

Nineteen patients were found to have AJCC stage II disease. The average DFS was 44 months in the low NTS group and 38 months in the high NTS group, but the difference was not significant (*p* = 0.23, Log rank test).

Expression of NTS in normal epithelium and expression of NTSR1 and NTSR3 were not significant risk factors for disease recurrence or all-cause mortality.

## 4. Discussion

This study is the first prospective cohort study into the protein expression of NTS, NTSR1, and NTSR3 in CRC. It involved the largest number of participants to date. It is the first to quantify the expressions of NTS as well as its receptors in human CRCs and associated surrounding normal colonic epithelium. Furthermore, this is novel in combining histopathological and clinical data to assess the association between the expression of NTS and its receptor with clinical outcomes.

This study demonstrated that NTS, NTSR1, and NTSR3 are detected in most colorectal cancer tissue. It further characterised the morphology of their expression. Quantification of staining gave further insights. Increased level of NTS were seen in more invasive cancers, as well as cancers which had lymphatic spread and EMVI. Finally, this study showed higher expression of NTS in cancer tissue was associated with worse DFS. These results, in line with previous studies, highlight the potential of the expression of NTS as a novel prognostic marker for clinical outcome in CRC.

This study found significantly higher levels of expression of NTS, NTSR1, and NTSR3 in cancer tissue compared to surrounding normal epithelium from the same individual. It also found positive correlations between the expression of NTS and NTSR3 with CRC pathological T stage. Of the 3 markers, NTSR1 showed the lowest expression levels overall. In contrast to findings of Gui et al., there was no significant correlation observed between cancer T stage and NTSR1 expression. This may be accounted by the different sensitivities of the techniques used (i.e., IHC to detect NTSR1 protein compared to in-situ hybridisation technique used to detect NTSR1 mRNA). Despite this, the findings corroborated that there was almost no NTSR1 protein expression in normal epithelium and higher expression in cancer tissue.

There appeared to be significant positive correlations between the expression levels of NTS, NTSR1, and NTSR3 in normal surrounding epithelium and cancer tissue taken from the same individual. This raises the question whether there might be a ‘field effect’ in some individuals where there is constitutive overexpression of NTS and its receptors creating a favourable milieu for CRC carcinogenesis, putting certain individuals at risk of developing CRC. It is also possible the over-expression of NTS and NTSR is a de novo feature acquired by the cancer cells during carcinogenesis. This area warrants further investigation through prospective cohort studies.

This study showed significant positive correlations between NTS and NTSR3 expression in cancer tissue. Moreover, there appeared to be a positive correlation between NTSR1 and NTSR3 expression. The role of NTSR3 is currently not well understood. It appears to be a sortilin receptor and are found in endoplasmic reticulum and cell membranes of CRC cells [24]. NTSR1 and NTSR3 forms a heterodimer on the surface of colon cancer cells. This complex is internalised upon stimulation by NTS and appears to modify the intracellular responses to NTS [25]. Membrane-associated form of NTSR3 also appear to be responsible for NTS endocytosis and transport to other destinations such as the nuclei where it could carry out mitogenic effects [26].

Lastly, this is the first study to show association of NTS expression and human CRC survival. Above average NTS expression in CRC tissue appeared to be a significant risk factor for shorter DFS. The results of this study highlights the potential of cancer NTS expression to be a prognostic marker for CRC. This clearly warrants further investigation.

The ability to identify patients with more aggressive disease and those who might benefit from adjuvant therapy is important in improving CRC survival. TNM staging remains the predominating factor. However, 10–20% of patients with AJCC stage 1 and 2 CRC who do not receive chemotherapy have disease progression during follow-up [13,14,16]. Personalisation of CRC treatment requires novel biomarkers which identify more aggressive cancers [27]. Currently, these include mutations in RAS, BRAF, CDX-2, as well as the presence of MSI. Routine evaluation of molecular markers has been endorsed by international guidelines and have become integral in the clinical management of CRC [28,29,30]. 

Bioinformatic analysis using the Gene Expression Omnibus database found NTS to be one of ten hub differentially expressed genes which predict cetuximab insensitivity in CRC [31]. In other cancers, bioinformatic studies using the Human Genome Atlas has identified NTS as a clinically significant differentially expressed gene in determining survival in oral squamous cell carcinoma [32]. NTSR1 mRNA expression appeared to be an important survival factor in smoking-associated lung adenocarcinoma [33]. Additionally, NTS has been identified as a hub differentially expressed gene in recurrence of lower-grade brain glioma [34]. It is important to carry out further studies to determine if CRC expression of NTS and NTSR are independent prognostic markers of cancer outcomes.

This study adds to the significant body of evidence for the role NTS and its receptors play in cancers [6,7,8,9,10,20,35]. For example, increased NTSR1 expression was associated with increased tumour size and number of metastatic lymph nodes in breast invasive ductal cell carcinomas [6]. NTSR1 expression was also elevated in human gastrointestinal stromal tumours [36]. Higher levels of NTS mRNA expression was found in higher grade serous ovarian carcinomas [37]. NTSR1 mRNA expression was higher in gastric cancers compared to non-cancerous tissue. In vitro evidence supported NTS induced activation of tumour growth and migration pathways which were mitigated by NTSR1 antagonisation with SR48692. The authors felt this was compelling evidence that NTSR1 could be a therapeutic target for gastric cancers [38].

The current study demonstrated over-expression of NTS, NTSR1, and NTSR3 in colorectal cancers compared to surrounding normal epithelium. Furthermore, higher NTS expression in CRC was also associated with higher grades of tumour invasion and worse disease-free survival. This raises the possibility that the NTS/NTSR axis could be a potential therapeutic target in CRC treatment. NTSR1 antagonists have been trialled in animal studies which demonstrated significant reduction in colorectal cancer growth in mice [39]. Other groups have shown that NTSR1 is a high potential target ligand in imaging and therapy for colonic, pancreatic and prostatic cancers [40,41,42]. Monoclonal antibodies against NTS appeared to restore sensitivity to cisplatin-based chemotherapy in human lung cancers xenografted into mice models with no obvious adverse effects [43]. Some advances have been made to translate these findings into clinical practice. NTSR1 targeted radioligand therapy was given to 6 patients with advanced pancreatic adenocarcinoma resulting in partial remission in some cases. Phase I/II clinical studies have already started (NCT03525392) [44]. The result of this study, along with past and future work, helps to build the case for trialling NTS/NTSR targeted therapy in CRC.

## 5. Limitations

This study included 58 participants with CRC. Despite being the largest and most detailed histological analysis to date, this limited the scope for further subgroup analysis. There were only 4 participants with metastatic disease. This is the result of the inclusion criteria of patients undergoing curative CRC resection. Larger cohorts are needed to investigate the generalisability of our findings. A major therapeutic dilemma in colorectal cancer is the identification of high-risk stage II cancers who require adjuvant treatment. In our study, only 19 patients had stage II disease. Although there appeared to be a difference in DFS between high NTS and low NTS subgroups (38 months vs 44 months respectively), the difference in survival was not statistically significant. This may partly be due to the small numbers of patients. Further large scales studies will allow stratification of data to evaluate the clinical utility of NTS and NTSR in colorectal cancer prognosis independent of established histological markers. The novel findings of this study highlight the need for this work and acts as the foundation for large scale studies following biomarker development roadmaps.

Within the study cohort, CDX2 was performed in 11 of the 58 patients. All 11 patients expressed CDX2. KRAS, NRAS, BRAF, and PI3KCA were only tested in two patients. Both patients had BRAF mutations but had wildtype for the other three markers which prevented meaningful analysis.

## 6. Conclusions

This clinical pathological study sheds light on the important association between NTS, its receptors and CRC. The expression of NTS in cancer appears to be a novel predictor of pathological and clinical outcome. This study, along with previously published work, which demonstrated significantly higher levels of plasma NTS in individuals with colorectal polyps and cancers compared to those without [45], highlights the potential for the neurotensinergic pathway to be exploited in the diagnosis and personalised treatment of CRC in the future.

## Figures and Tables

**Table 1:** Participant demographics and treatment details.

	Median	IQR	
**Age (years)**	72.2	63.9–82.4	
**BMI**	26.9	24.4–29.9	
	**Category**	**Number**	**Frequency %**
**Sex**	Male	30	51.7
	Female	28	48.3
**Ethnicity**	Black	6	10.3
	East Asian	2	3.4
	South Asian	5	8.6
	White	38	65.5
	Other	7	12.1
**American Society of**	1	13	22.4
**Anaesthesiologists**	2	17	29.3
**physical status**	3	28	48.3
	4	0	0
**American Joint**	I	22	37.9
**Committee on Cancer**	II	19	32.8
**Clinical Stage**	III	13	22.4
	IV	4	6.9
**Length of Hospital Stay** **(Days)**	**Median** 7.0	**IQR** 5.0–10.5	
**Perioperative Carcinoembryonic antigen**	2.0	1.0–4.0	
		**Number**	**Frequency (%)**
**Operation**	Right Hemicolectomy	21	36.2
	Extended Right Hemicolectomy	6	10.3
	Left Hemicolectomy	4	6.9
	Sigmoid Colectomy	3	5.2
	Anterior Resection	16	27.6
	Abdominal Perineal Excision	4	6.9
	Appendicectomy	1	1.7
	Transanal endoscopic mucosal resection	1	1.7
	Hartmann’s Resection	2	3.4
**Laparoscopic**	Open	11	19.0
	Laparoscopic	39	67.2
	Converted to Open	8	13.8
**Complications**	No	46	79.3
	Yes	12	20.7
**Clavien-Dindo Grade**	0	46	79.3
	1	0	0
	2	1	15.5
	3	1	1.7
	4	1	1.7
	5	1	1.7
**Anastomotic Leak**	No	55	94.8
	Yes	1	1.7
	N/A (Transanal endoscopic mucosal resection/Appendicectomy)	2	3.4
**Neoadjuvant Therapy**	No	55	94.8
	Yes	2	3.4
	Missing	1	1.7
**Adjuvant Therapy**	No	38	65.5
	Yes	19	32.7
	Missing	1	1.7

**Table 2:** Clinical pathological characteristics of colorectal cancers.

	Median		IQR
**Number of Lymph Nodes Harvested**	24.5		18.5–32.0
**Cancer Maximum Diameter (mm)**	35.0		20.0–47.0
**Rectal Cancer Height from Anal Verge (mm)**	90.0		67.0–110.0
		**Number**	**Frequency (%)**
**Location of Cancer**	Caecum	12	20.7
	Ascending Colon	9	15.5
	Transverse Colon	4	6.9
	Splenic Flexure	2	3.4
	Descending Colon	1	1.7
	Sigmoid Colon	9	15.5
	Rectum	18	31.0
	Appendix	1	1.7
	Multiple sites	2	3.4
**Grade of Differentiation**	Well Differentiated	2	3.4
	Moderately Differentiated	47	81.0
	Poorly Differentiated	8	13.8
	Missing	1	1.7
**Background of Serrated Adenoma**	Yes	3	5.2
	No	55	94.8
**Seen to arising from polyp**	Yes	16	27.6
	No	42	72.4
**Mucinous Cancer**	Yes	19 (1 signet ring cancer)	32.7
	No	39	67.2
**Extra Mural Vascular Invasion**	Yes	11	19.0
	No	48	79.3
	Missing	1	1.7
**Lymphovascular Invasion**	Yes	18	31.0
	No	39	67.2
	Missing	1	1.7
**Perineural Invasion**	Yes	7	12.1
	No	49	84.5
	Missing	2	3.4
**Tumour Budding**	None	11	19
	Minimal/Focal	16	27.6
	Present	16	27.6
	Prominent	12	20.7
**Lymphoid Response**	None	12.1	13.0
	Mild	27.6	29.6
	Moderate	15.5	16.7
	Prominent	1.3	11.1
	Brisk—Crohn’s Like Response	27.6	29.6
**Pathological T-stage**	1	8	13.8
	2	16	27.6
	3	21	36.2
	4	13	22.4
**Pathological N-Stage**	0	41	70.7
	1	11	19.0
	2	6	10.3
**Resection Margin**	0	54	93.1
	1	4	6.9
	2	0	0
**M Stage**	0	54	93.1
	1	3	5.2
	2	1	1.7
**Microsatellite Instability**	Present	6	10.3
	Not Present	20	34.5
	Not done	32	55.2

**Table 3:** H-scores of expressions of NTS, NTSR1 and NTSR3 across different cancer T stages. * Independent sample Kruskal-Wallis Test.

	T1 (8)	T2 (16)	T3 (21)	T4 (13)	*p*
**NTS Cancer Tissue**					
Median	115.25	157.6	163.9	189.8	<0.01 *
IQR	78.7–160.9	109.1–173.6	131.6–176.3	168.1–198.1	
Range	21.0–178.3	37.0–182.5	95.5–198.3	143.0–240.0	
**NTSR1 Cancer Tissue**					
Median	1.1	3.6	8.8	12.5	0.45 *
IQR	0–13.5	2.2–24.7	0.0–24.1	0.0–24.6	
Range	0–16.3	0.0–45.0	0.0–64.7	0.0–74.5	
**NTSR3 Cancer Tissue**					
Median	8.9	34.9	34.4	46.8	<0.01 *
IQR	1.4–21.9	17.8–82.6	25.0–57.4	37.1–104.1	
Range	0.0–54.9	13.3–105.5	19.3–151.1	25.0–175.5	
	**T1**	**T2**	**T3**	**T4**	***p***
**NTS Normal Epithelium**					
Median	27.5	94.0	125.0	120.4	0.22 *
IQR	21.3–82.8	47.0–130.2	75.0–149.1	82.7–161.5	
Range	12.5–155.0	22.5–173.3	25.0–175.5	42.1–175.0	
**NTSR1 Normal Epithelium**					
Median	0.0	0.00	0.0	0.0	0.98 *
IQR	0.0–0.0	0.0–0.0	0.0–0.0	0.0–0.0	
Range	0.0–4.5	0.0–7.5	0.0–21.4	0.0–3.5	
**NTSR3 Normal Epithelium**					
Median	0.0	24.8	24.3	31.9	0.11 *
IQR	0.0–9.4	10.9–54.1	9.4–31.8	14.5–73.2	
Range	0.0–34.25	0–87.5	0.0–162.5	0.0–145.7	
